# Morphological Shifts of the External Flight Apparatus across the Range of a Passerine (Northern Wheatear) with Diverging Migratory Behaviour

**DOI:** 10.1371/journal.pone.0018732

**Published:** 2011-04-18

**Authors:** Marc I. Förschler, Franz Bairlein

**Affiliations:** Institute of Avian Research, Vogelwarte Helgoland, Wilhelmshaven, Germany; Raymond M. Alf Museum of Paleontology, United States of America

## Abstract

We studied morphological differentiation in the flight apparatus of the four currently recognised sub-species of Northern Wheatears, *Oenanthe oenanthe*. Considering all measured birds without assigning them a priori to any sub-species we found a clinal morphological shift. Relative wing length, wing pointedness, and the degree of tail forking were positively correlated with migratory distance, whereas tail length (relative to wing length) was negatively correlated. The large-sized, long-distance migrant “Greenland” Wheatear, *O. o. leucorhoa*, is characterized by relatively longer, broader and more pointed wings and more forked tails, similar to the smaller-sized nominate Northern Wheatear, *O. o. oenanthe*, from North Europe, Siberia and Russia. In contrast, the short distance migrant “Seebohm's” Wheatear, *O. o. seebohmi*, from northwest Africa, possesses much rounder wings, and the tail is relatively longer and less forked. Sub-species with intermediate migratory habits (different populations of nominate Northern Wheatear, *O. o. oenanthe*, and “Mediterranean” Northern Wheatear, *O. o. libanotica*) show, as expected, intermediate features according to their intermediate migratory behaviour. Our results are congruent with other inter- and intraspecific studies finding similar adaptations for energy-effective flight in relation to migration distance (morphological migratory syndrome).

## Introduction

The morphology of the avian wing constitutes a trade-off between various selection pressures that act on its aerodynamic and mechanical properties [1]–[3]. The evolution of wing and tail size and shape is affected by the diverging demands of migratory behaviour, take-off ability in response to predator attacks and by the density of obstacles that constrain flight manoeuvrability in the occupied habitats [4]–[7].

Slender and more pointed wings and shorter tails in relation to the wing reduce the induced drag at the wings considerably and are known to produce a larger forward component in flight during migration [1]–[3], [8]. Furthermore, more forked tails are known to provide higher uplift and lower drag [3], [9]. Consequently, we may assume that the extent of migratory behaviour results in changes in the external morphology of the flight apparatus [10]–[12] which select for energy-efficient flight [13].

Many studies have shown that wing pointedness correlates with migratory behaviour, also known as “Seebohm's rule” [4]–[5], [7], [13]–[18]. In a general approach across several taxa, Leisler & Winkler [11] established the generalisation that migrants have relatively longer and more pointed wings and also higher aspect ratios. This pattern has been repeatedly confirmed at the intraspecific level [13], [19]–[26]. Among different populations of blackcaps, *Sylvia atricapilla*, Fiedler [13] found with increasing migratory distance: (1) an increase in wing length, aspect ratio and wing pointedness; (2) a decrease in wing-load; (3) relatively shorter slots on the wing-tip; (4) a shorter alula in relation to wing length; and (5) a shorter tail in relation to wing length. These changes were significantly greater than expected from the simple trend of increasing body mass from southern to northern populations [13] and evolved obviously under the demands of diverging migratory behaviour.

**Figure 1:**
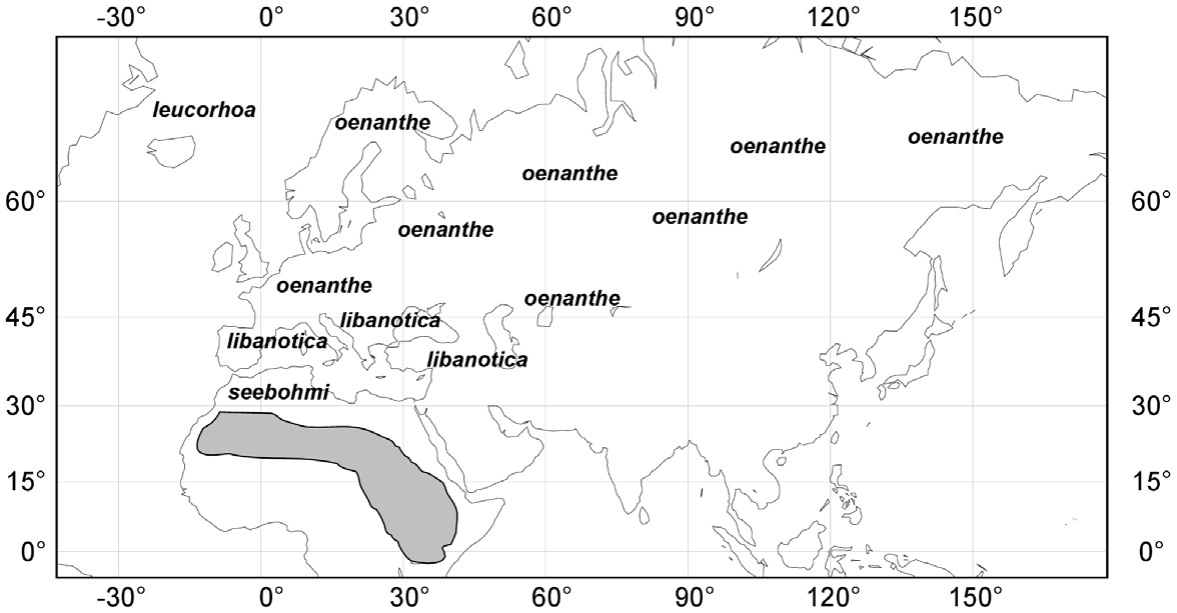
Breeding distribution range of the four sub-species of the Northern Wheatear *Oenanthe oenanthe* and their wintering area [5]. The species has one of the largest breeding ranges for a passerine. The whole population winters in sub-Saharan Africa (in grey; [33]).

The Northern Wheatear, *Oenanthe oenanthe* (Linnaeus, 1785), is one of the most diverse migratory song birds of the Palaearctic and therefore well suited for an intra-specific study [27]. This species is distributed from North Africa northwards to Iceland and Greenland and continuously from Europe towards eastern Russia [28]. Small populations have even settled the Nearctic region (Canada and Alaska). All populations still overwinter in sub-Saharan Africa and need to migrate large distances in order to reach their winter quarters. However, the distinct populations differ considerably in the distances they have to travel (Figure 1).

Four sub-species of Northern Wheatears are currently recognised [28]. The sub-species *O. o. seebohmi* (Dixon, 1882; “Seebohm's” Wheatear) is restricted to the Atlas mountains of northwest Africa. The male nuptial plumage of this form is quite distinct from all other Northern Wheatears, and it is therefore sometimes treated as a separate species [28]. It shows the shortest migration distances, wintering mainly in southwestern Mauritania and Senegal [29]. The sub-species *O. o. libanotica* (Hemprich & Ehrenberg, 1833; “Mediterranean” Northern Wheatear) is continuously distributed from southern Europe eastwards over Asia Minor, and Transcaucasia to Mongolia and China [28]. These birds winter in Mesopotamia and the northern Afrotropics. The sub-species *O. o. oenanthe* (Linnaeus 1758; nominate Northern Wheatear) shows the largest range inhabiting the whole of northern and central Europe, north Asia to eastern Siberia and the northwestern parts of North America (Alaska and Northwest Canada). The wintering grounds of this sub-species are situated in central Africa. The sub-species *O. o. leucorhoa* (Gmelin 1789, “Greenland” Northern Wheatear), finally, is found in Greenland, Iceland, the Faroe Islands and in northeastern Canada, and it winters in western Africa [28]. Several other named sub-species are currently not recognised as taxonomic entities: *O. o. rostrata* (Hemprich and Ehrenberg, 1833; from Mesopotamia, eastern Egypt, northern Arabia, Syria, Caucasus), *O. o. nivea* (Weigold, 1913; southern Spain, Balearic Islands) and *O. o. virago* (Meinertzhagen, 1920; islands of eastern and southern Aegean, southeastern Europe) regarded as synonyms of *O. o. libanotica; O. o. argentea* (Lonnberg, 1909; Transbaikal), regarded as a synonym of *O. o. oenanthe;* and *O. o. schiöleri* (Salomonsen, 1927; Island, Färöer), a synonym of *O. o. leucorhoa*. The assignment of populations to the sub-species *O. o. oenanthe* and *O. o. libanotica* remains to some extent arbitrary, because the geographical limits of both forms have not been well studied. The Somali Wheatear, *O. phillipsi* (Shelley, 1885; Somalia and Ethiopia), has been treated formerly as another sub-species of the Northern Wheatear, but recent genetic studies show that this form is a distinct species [30]–[31].

The wide distribution of the populations of Northern Wheatears suggests specific adaptations to migration, depending on the distance the birds have to travel [27]. We therefore studied museum specimens to examine how different migratory behaviours correlate with the morphologies of the different subspecies. In particular, we studied which morphological changes of the external flight apparatus are directly linked to the differences in migratory distances, and if a general morphological migratory syndrome exists which evolved under the constraints of diverging needs for the adaptation to migration.

## Results

**Figure 2:**
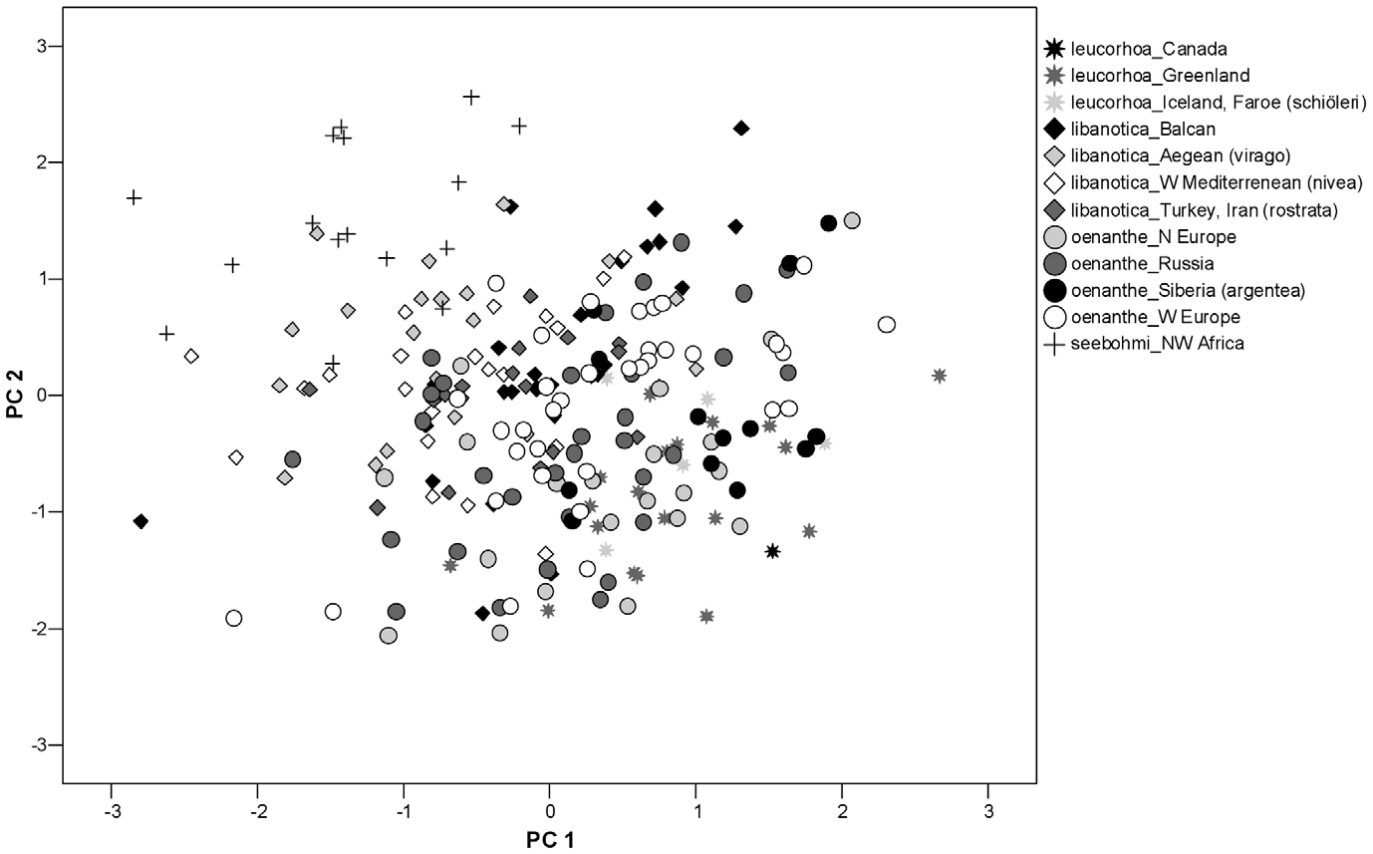
PC1-PC2 plane of PCA performed on 9 morphometric variables of the flight apparatus measured in several populations of the four sub-species of Northern Wheatear *Oenanthe oenanthe*. For PC1 the loadings were considerably stronger in WL, ATWT, P1Wt, S1Wt, and for PC2 in TF, TL, WW, NoP2, NoP3 (compare Table 4).

The four currently recognised sub-species of the Northern Wheatear show clear morphological differentiation in the flight apparatus (Table 1). Our ANOVAs identified various significant differences both in uncorrected and body size corrected analysis (Table 2, Table 3). Using a PCA on the 9 morphometric variables of the flight apparatus (size corrected; log-transformed; varimax rotation) we obtained two relevant principal components (PCs) with an Eigen-Value >1 explaining 62.6% of total variance (Table 4, Figure 2). PC1 explained 38.9% of the total variance and comprises wing length (maximal wing chord), the distance of first secondary-wing tip, distal primary-wing tip and alula-wing tip. PC2 explained 23.7% of variance and comprises tail length, the strength of the fork of the tail, wing width and the notches of P2 and P3. A statistical comparison of PC1 between the four subspecies showed clear differentiations (ANOVA: F_3,241_ =  31.27; p<0.001), with all four groups being significantly different from each other (Bonferroni correction, p<0.05). The same holds for PC 2 (ANOVA, F_3,241_ =  36.77; p<0.001) with all four groups being significantly different from each other with the exception of *O. o. libanotica* versus *O. o. leucorhoa* (Bonferroni correction, p<0.05).

**Table 1:** Values for 9 morphometric variables and 2 calculated indices of the flight apparatus in the four sub-species of Northern Wheatear *Oenanthe oenanthe*.

Variable	*leucorhoa* (n = 24)	*oenanthe* (n = 106)	*libanotica* (n = 94)	*seebohmi* (n = 18)
WL	102.54±0.48	96.19±0.26	94.17±0.29	95.11±0.76
WW	68.88±0.41	64.97±0.17	64.95±0.20	68.19±0.43
S1Wt	33.69±0.26	31.00±0.18	28.78±0.17	26.03±0.31
P1Wt	62.79±0.42	57.98±0.25	56.27±0.26	54.69±0.39
AtWt	75.48±0.36	69.81±0.26	68.83±0.25	68.19±0.48
NoP2	24.94±0.19	23.97±0.13	24.21±0.14	25.97±0.25
NoP3	29.04±0.22	28.59±0.16	28.96±0.16	30.50±0.34
TL	58.27±0.51	55.69±0.23	56.24±0.22	58.70±0.53
TF	−3.54±0.16	−3.47±0.09	−2.77±0.10	−0.92±0.35
Tail-wing ratio	56.85±0.54	57.87±0.21	59.76±0.26	61.20±0.54
Wing shape index	0.29±0.003	0.27±0.002	0.25±0.002	0.22±0.004

Given are the means with standard error (SE).

**Table 2:** Comparison of 9 morphometric variables of the flight apparatus in the four sub-species of Northern Wheatear *Oenanthe oenanthe*.

Variable	uncorrected		body size corrected	
	ANOVA	Bonferroni	ANOVA	Bonferroni
WL	F_3,239_ = 60.20;	**leu vs oen (P<0.001)**	F_3,234_ = 5.43;	leu vs oen (P = 1.000)
	P<0.001	**leu vs lib (P<0.001)**	P = 0.001	**leu vs lib (P = 0.019)**
		**leu vs see (P<0.001)**		leu vs see (P = 0.200)
		**oen vs lib (P<0.001)**		**oen vs lib (P = 0.007)**
		oen vs see (P = 0.735)		oen vs see (P = 0.451)
		lib vs see (P = 1.000)		lib vs see (P = 1.000)
WW	F_3,237_ = 45.50;	**leu vs oen (P<0.001)**	F_3,235_ = 4.29;	leu vs oen (P = 1.000)
	P<0.001	**leu vs lib (P<0.001)**	P = 0.006	leu vs lib (P = 1.000)
		leu vs see (P = 1.000)		**leu vs see (P = 0.044)**
		oen vs lib (P = 1.000)		oen vs lib (P = 1.000)
		**oen vs see (P<0.001)**		**oen vs see (P = 0.003)**
		**lib vs see (P<0.001)**		**lib vs see (P = 0.006)**
S1Wt	F_3,238_ = 100.19;	**leu vs oen (P<0.001)**	F_3,236_ = 64.60;	leu vs oen (P = 0.187)
	P<0.001	**leu vs lib (P<0.001)**	P<0.001	**leu vs lib (P<0.001)**
		**leu vs see (P<0.001)**		**leu vs see (P<0.001)**
		**oen vs lib (P<0.001)**		**oen vs lib (P<0.001)**
		**oen vs see (P<0.001)**		**oen vs see (P<0.001)**
		**lib vs see (P<0.001)**		**lib vs see (P<0.001)**
P1Wt	F3,238 = 54.47;	**leu vs oen (P<0.001)**	F_3,236_ = 14.01;	leu vs oen (P = 0.205)
	P<0.001	**leu vs lib (P<0.001)**	P<0.001	**leu vs lib (P<0.001)**
		**leu vs see (P<0.001)**		**leu vs see (P<0.001)**
		**oen vs lib (P<0.001)**		**oen vs lib (P = 0.002)**
		**oen vs see (P<0.001)**		**oen vs see (P<0.001)**
		lib vs see (P = 0.078)		**lib vs see (P = 0.044)**
AtWt	F_3,238_ = 48.82;	**leu vs oen (P<0.001)**	F_3,236_ = 5.62;	leu vs oen (P = 0.225)
	P<0.001	**leu vs lib (P<0.001)**	P = 0.001	**leu vs lib (P = 0.009)**
		**leu vs see (P<0.001)**		**leu vs see (P = 0.002)**
		**oen vs lib (P = 0.032)**		oen vs lib (P = 0.417)
		oen vs see (P = 0.062)		oen vs see (P = 0.066)
		lib vs see (P = 1.00)		lib vs see (P = 0.718)
NoP2	F_3,238_ = 13.91;	**leu vs oen (P = 0.006)**	F_3,234_ = 7.37;	leu vs oen (P = 1.000)
	P<0.001	leu vs lib (P = 0.086)	P<0.001	leu vs lib (P = 0.289)
		leu vs see (P = 0.073)		**leu vs see (P<0.001)**
		oen vs lib (P = 1.000)		oen vs lib (P = 0.812)
		**oen vs see (P = 0.001)**		**oen vs see (P<0.001)**
		**lib vs see (P<0.001)**		**lib vs see (P = 0.004)**
NoP3	F_3,236_ = 7.90;	leu vs oen (P = 1.000)	F_3,239_ = 8.94;	**leu vs oen (P = 0.041)**
	P<0.001	leu vs lib (P = 1.000)	P<0.001	**leu vs lib (P<0.001)**
		**leu vs see (P = 0.017)**		**leu vs see (P<0.001)**
		oen vs lib (P = 0.593)		oen vs lib (P = 0.374)
		**oen vs see (P<0.001)**		**oen vs see (P = 0.004)**
		**lib vs see (P = 0.001)**		lib vs see (P = 0.103)
TL	F_3,238_ = 14.68;	**leu vs oen (P<0.001)**	F_3,236_ = 5.07;	leu vs oen (P<1.000)
	P<0.001	**leu vs lib (P = 0.001)**	P = 0.002	leu vs lib (P = 0.308)
		leu vs see (P = 1.000)		**leu vs see (P = 0.004)**
		oen vs lib (P = 0.565)		oen vs lib (P = 0.604)
		**oen vs see (P<0.001)**		**oen vs see (P = 0.005)**
		**lib vs see (P = 0.001)**		lib vs see (P = 0.089)
TF	F_3,238_ = 38.20;	leu vs oen (P = 1.000)	F_3,236_ = 33.69;	leu vs oen (P = 0.393)
	P<0.001	**leu vs lib (P = 0.005)**	P<0.001	**leu vs lib (P = 0.005)**
		**leu vs see (P<0.001)**		**leu vs see (P<0.001)**
		**oen vs lib (P<0.001)**		**oen vs lib (P<0.001)**
		**oen vs see (P<0.001)**		**oen vs see (P<0.001)**
		**lib vs see (P<0.001)**		**lib vs see (P<0.001)**

Results are shown for both uncorrected and body size corrected values (divided by tarsus length). Given are the F-values with degrees of freedom, significance and Bonferroni pairwise comparison. Significant differences (P<0.05) are highlighted in bold.

**Table 3:** Comparison of 2 indices of the flight apparatus in the four sub-species of Northern Wheatear *Oenanthe oenanthe*.

Variable	ANOVA	Bonferroni
Tail-wing ratio	F_3,239_ = 25.78;	leu vs oen (P = 0.338)
	P<0.001	**leu vs lib (P<0.001)**
		**leu vs see (P<0.001)**
		**oen vs lib (P<0.001)**
		**oen vs see (P<0.001)**
		**lib vs see (P = 0.007)**
Wing shape index	F_3,230_ = 62.79;	**leu vs oen (P = 0.002)**
	P<0.001	**leu vs lib (P<0.001)**
		**leu vs see (P<0.001)**
		**oen vs lib (P<0.001)**
		**oen vs see (P<0.001)**
		**lib vs see (P<0.001)**

Given are the F-values with degrees of freedom, significance and Bonferroni pairwise comparison. Significant differences (P<0.05) are highlighted in bold.

**Table 4:** Loadings of the PCA performed on 9 morphometric variables of the flight apparatus measured in the four sub-species of Northern Wheatear *Oenanthe oenanthe* (Varimax-rotation with Kaiser-Normalisation).

Variable	PC 1	PC 2
WL	0.847	0.399
TL	0.366	0.697
TF	−0.303	0.678
S1Wt	0.861	−0.217
WW	0.466	0.676
P1Wt	0.891	0.192
AtWt	0.849	0.327
NoP2	0.148	0.832
NoP3	0.184	0.754

**Figure 3:**
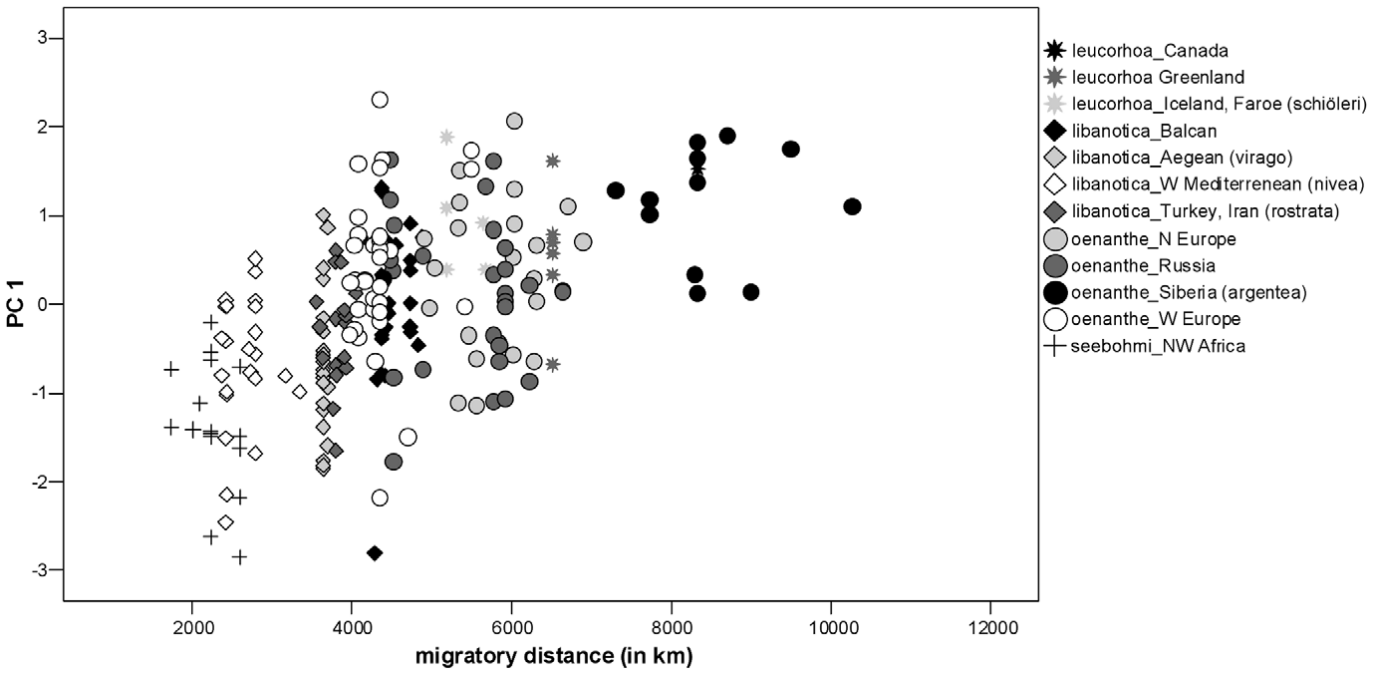
Relationship between PC1 (WL, AtWT, P1Wt, S1Wt) and migratory distance. Populations of Northern Wheatears with longer migration pathways have relatively longer wings (for statistics, see Table 5).

**Figure 4:**
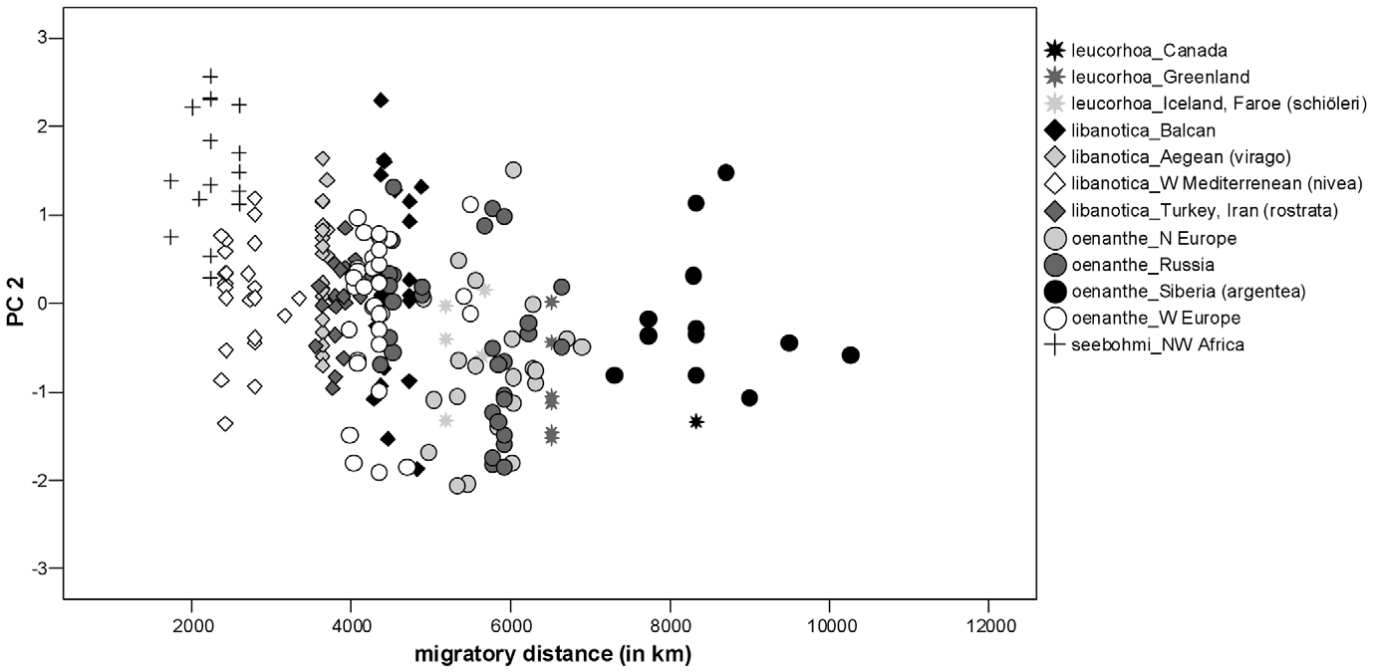
Relationship between PC2 (TF, TL, WW, NoP2, NoP3) and migratory distance. Populations of Northern Wheatears with longer migration pathways have more narrow wings, relatively shorter and stronger forked tails and shorter emarginations in the primaries (for statistics, see Table 5).

**Figure 5:**
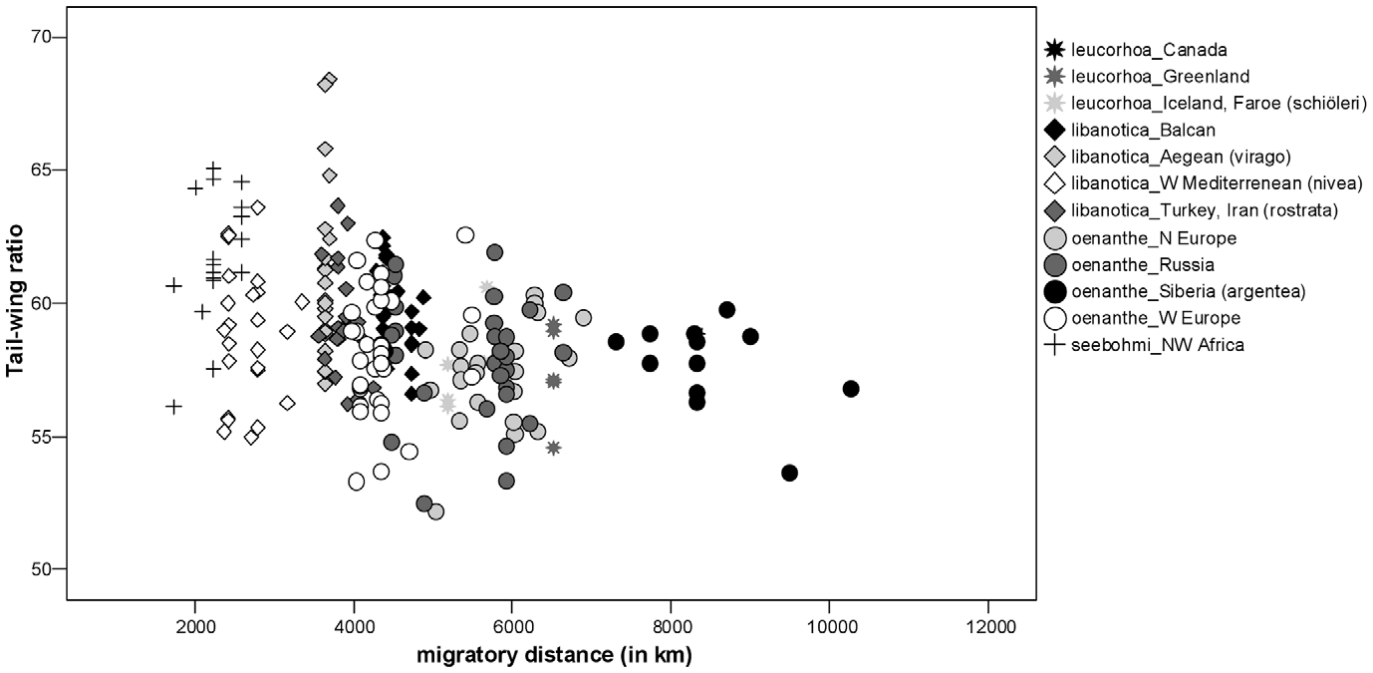
Relationship between tail-wing ratio and migratory distance. Populations of Northern Wheatears with longer migration pathways have shorter tails in relation to wing length (for statistics, see Table 5).

**Figure 6:**
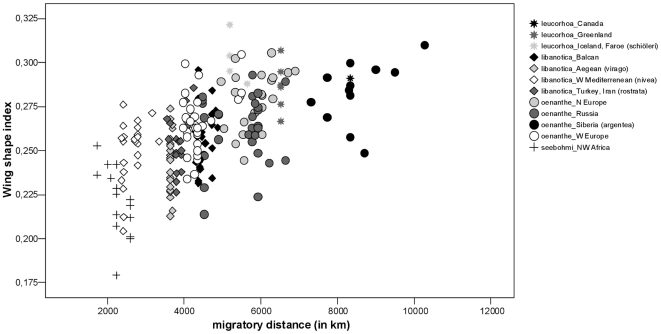
Relationship between wing pointedness (wing shape index [13]) and migratory distance. Populations of Northern Wheatears with longer migration pathways have more pointed wings (for statistics, see Table 5).

Since obvious clinal variation exists within the single sub-species of the Northern Wheatear and the separation of the sub-species is not always accurate due to distribution overlap, we conducted linear regressions independent of taxonomic status. In these analyses we included only specimens for which we had details on the collection localities (n = 234). We found a significant correlation between both principal components (PC1, PC2) and the migratory distance (Table 5, Figures 3 and 4). Birds with longer migratory pathways had (1) relative longer (WL) and more pointed wings (S1Wt); (2) relatively more narrow wings (WW); (3) a shorter alula and P1 in relation to wing length (AtWt, P1Wt); (4) relatively shorter emarginations on the wing-tip (NoP2, NoP3); and (5) relatively shorter and more forked tails in relation to wing length (TL, TF). Regressions of migratory distance with tail-wing ratio and wing shape index revealed congruent results (Table 5). Birds with longer migration distances showed relatively shorter tails in relation to wing length (Figure 5) and more pointed wings (Figure 6).

**Table 5:** Regression analyses between migratory distance and PC1 (WL, ATWT, P1Wt, S1Wt), PC2 (TF, TL, WW, NoP2, NoP3.), tail-wing ratio, and wing shape index in Northern Wheatear *Oenanthe oenanthe* (n = 235).

Variable	R	df	F	P
PC1	0.543	223	92.928	P<0.001
PC2	0.412	223	45.431	P<0.001
Tail-wing ratio	0.336	230	29.099	P<0.001
Wing shape index	0.631	221	145.47	P<0.001

## Discussion

Our results show that different populations of the four currently recognised sub-species of the Northern Wheatear (*O. o. leucorhoa, O. o. oenanthe, O. o. libanotica, O. o. seebohmi*) are strongly differentiated in several morphometric characteristics of their flight apparatus. A regression analyses independent of taxonomic status revealed that the flight apparatus of Northern Wheatears has been shaped along a phenotypic continuum, obviously according to the extent of the conducted migratory movements. Birds with longer migratory pathways possess relatively longer, more pointed, and more slender wings, shorter emarginations on the wing tip, and show relatively shorter tails in relation to wing length and a more forked tail.

The large sub-species *O. o. leucorhoa* shows the strongest adaptations to long-distance migration, because it is the only form which needs to cross a large water body (north Atlantic) during migration. These adaptations include relative longer, broader and more pointed wings and stronger forked tails, which may help to stabilise the bird during migration in harsh climatic conditions over the sea. Similar results were obtained in a recent study by Delingat and colleagues [27], who showed by means of isotopic analyses that presumed Greenlandic Northern Wheatears of the sub-species *O. o. leucorhoa* have more pointed wings than their congeners from other European breeding areas. However, in our study we found that, despite smaller size, *O. o. oenanthe* from Siberia, North Europe and Russia have very similar adaptations in the flight apparatus in relation to the long distance these birds have to travel. The other extreme of the Northern Wheatears, the sub-species *O. o. seebohmi* from North Africa, which only crosses the comparatively short distance over the Sahara to winter in west Africa, has a much rounder wing and the tail is considerably less forked. Western *O. o. oenanthe* and the members of the sub-species *O. o. libanotica* show intermediate features and overlap in their morphology according to the migratory distance they have to travel. *O. o. libanotica* of the western Mediterranean (formerly sub-species *O. o. nivea*) and the Aegean (formerly sub-species *O. o. virago*) with short migration distances are morphologically more similar to the birds of the sub-species *O. o. seebohmi*, while *O. o. oenanthe* from West Europe are morphologically more similar to *O. o. libanotica* from the Balkan and Turkey, Iran (formerly sub-species *O. o. rostrata*).

The adaptations in the flight apparatus observed in our study follow the general predictions of the so-called migratory syndrome [32]. Similar to the study of Fiedler [13], we found birds with a more “migratory type” flight apparatus to have developed a more efficient morphology of the external flight apparatus than their less migratory conspecifics. Studies on aerodynamics of bird flight [1], [2], [8] have demonstrated that the observed morphological shift with increasing migratory distances is well suited to produce a larger forward component in flight due to a more prominent distal part of the wing. The more slender and pointed wings and the shorter tail in relation to the wing reduce the induced drag at the wings and produce greater uplift and thrust [2], [3]. Additionally, the short and stronger forked tails provide high lift and low drag [3], [9].

As a possible trade-off, the adaptations for migration constrain the manoeuvrability of the birds. A decrease of Reynolds number due to a higher aspect ratio of the wing and a reduced ability of the tips to bend and generate lift due to relatively short notches at the wing tip result in a reduced capacity for very slow flights under high angles of attack [2], [13]. Additionally, relatively short tails generate less lift in slow flights and reduce the ability of the tail to start or stop roll manoeuvres [9].

Besides, it is likely that other factors might have influenced the morphological differentiation of the flight apparatus as well, such as differences in foraging, breeding habitat or sexual selection of different sub-populations. However, because all sub-species live in very similar habitat types (open, rocky areas) and show equivalent breeding and foraging behaviour, we believe that the demands for migration are the main driving forces for the morphological shift of the flight apparatus.

To summarize, the intraspecific patterns in flight apparatus that we found in the Northern Wheatear nicely follow the expectations drawn from other work [11], [13], [32], indicating that in birds travelling longer distances the traits for energy-effective flight (in terms of distance travelled per energy expended) are obviously more strongly developed then the traits for manoeuvrability. Future work needs to reveal how these changes in external flight morphology are linked to other physiological, behavioural and internal morphological adaptations to migration and how fast these morphological shifts may appear in the evolutionary history of a species.

## Materials and Methods

We measured external morphological traits of the flight apparatus to compare between Northern Wheatears of the four currently recognised sub-species with different migratory behaviour (Figure 1). Specimens from the following European museum collections were used (Appendix S1): Zoologisches Forschungsmuseum Alexander König (Bonn), Senckenberg Museum (Frankfurt), Muséum National d'Histoire Naturelle (Paris), Natural History Museum (Tring), Zoologische Staatssammlung (Munich), Museum für Tierkunde (Dresden), Staatliches Museum für Naturkunde (Stuttgart), Biozentrum Grindel and Zoologisches Museum (Hamburg), Überseemuseum (Bremen) and Institut für Vogelforschung “Vogelwarte Helgoland” (Wilhelmshaven).

Nine external characters of the flight apparatus were measured to the nearest 0.1 mm [11] (Table 6). Furthermore, we calculated tail-wing ratio and wing shape index. The latter was derived by the following formula: Wing shape index  =  (differences between longest primary and innermost primary – difference between longest primary and outermost primary)/wing length following Fiedler [13]. A higher value indicates a more pointed wing. In order to guarantee comparability between specimens we used only skins of adult male specimens in spring or summer plumage collected from breeding areas. We calculated the distance between collection place and main wintering area [33] following the method of Imboden & Imboden [34].

**Table 6:** The 9 morphometric variables (measured) as defined by Leisler & Winkler [11] and the two indices used in our study.

WL	maximum wing chord
WW	wing width, distance between carpal joint and tip of the longest secondary
S1Wt	first secondary to the wing tip (the tip of the longest primary)
P1Wt	first tiny primary to the wing tip (the tip of the longest primary)
AtWt	tip of the longest alula to the wing tip (the tip of the longest primary)
NoP2	length of the notch on the inner web of the second primary
NoP3	length of the notch on the outer web of the third primary
TL	tail length, from insertion of central pair of feathers to tip of longest rectrix
TF	fork of the tail, distance from the longest to the shortest rectrix (negative)
Tail-wing ratio	ratio of tail length to maximum wing chord
Wing shape index	wing pointedness [13]

Primaries are numbered from the outermost to the innermost.

In total, we obtained data from 242 male Northern Wheatears, *Oenanthe oenanthe*. For general comparison of the four currently recognised sub-species (*O. o. oenanthe,* n = 106, *O. o. libanotica,* n =  94*; O. o. seebohmi,* n = 18, *O. o. leucorhoa*, n = 24), we conducted ANOVAs for the two indices and each of the nine parameters both uncorrected and corrected for body size (divided by tarsus length). Because several of the nine original variables of the flight apparatus were correlated with each other, we subsequently conducted a principal component analysis (PCA) including the size corrected values of all variables. In order to account for allometry and to normalize distribution, we log-transformed all measurements.

Finally we conducted regression analyses between migratory distance and the two principal components and the two indices (the latter two showed normal distribution). We did not correct for phylogeny, because all forms are closely related and currently no comprehensive tree exists for the genetic relationship between the different sub-populations and sub-species. All analyses were done in SPSS 12.0.

## Supporting Information

Appendix S1 Specimens of Northern Wheatear *Oenanthe oenanthe* measured in different museums. Given are the collection numbers and the assigned sub-species group.
(DOC)
