# Supplementary material for: Morphological Shifts of the External Flight Apparatus across the Range of a Passerine (Northern Wheatear) with Diverging Migratory Behaviour
Source: PLoS One. 2011 Apr 18;6(4):e18732. doi: 10.1371/journal.pone.0018732 (PMC3078915; doi:10.1371/journal.pone.0018732)
Supplement: Appendix S1 — Specimens of Northern Wheatear Oenanthe oenanthe measured in different museums. Given are the collection numbers and the assigned sub-species group. (DOC) [file pone.0018732.s001.doc]

**Table S1.** Specimens of Northern Wheatear *Oenanthe oenanthe* measured in different museums. Given are the collection numbers and the assigned sub-species group.

|  | *O.o.leucorhoa* | *O.o.oenanthe* | *O.o.libanotica* | *O.o.seebohmi* |
| --- | --- | --- | --- | --- |
| museum | *n=24* | *n=107* | *n=93* | *n=18* |
| Berlin | 26176  26178 | 70631  70632  74483  b436  b436  b436  b454  b563_12 | 3726  3727  3735  27725  27726  27727  251455  251458  301184  200.011.521  b1050  b1051  b489_10  b910_34  b910_36  b910_37  b910_40 | 1119  1131  70425 |
| Bonn | 234  571.572  H II 16  H II 16  Kleinschmidt 2397  Kleinschmidt 2398  Kleinschmidt 2406 | 2457  2458  2459  2853  50.384  50.385  79.315  79.316  79.317  79.318  571.567  H II 16  H II 16  H II 16  H II 16  Kleinschmidt 2407  Kleinschmidt 2408  Kleinschmidt 2409  Kleinschmidt 2411  Kleinschmidt 2412  Kleinschmidt 2413  Kleinschmidt 2422  Kleinschmidt 2423  Kleinschmidt 2434  Kleinschmidt 2436  Kleinschmidt 2437  Kleinschmidt 2438  Kleinschmidt 2439  Kleinschmidt 2440  Kleinschmidt 2441  Kleinschmidt 2442  Kleinschmidt 2443  Kleinschmidt 2444  Kleinschmidt 2445  Kleinschmidt 2446  Kleinschmidt 2447  Kleinschmidt 2478  Kleinschmidt 2479  Kleinschmidt 2486  Kleinschmidt 2487  Kleinschmidt 2488  Kleinschmidt 2491  Kleinschmidt 2492  Kleinschmidt 2494  Kleinschmidt 2498  Kleinschmidt 2499  Kleinschmidt 2500  Kleinschmidt 2501  Kleinschmidt 2502  Kleinschmidt 2504  Kleinschmidt 2505 | 38.821  38.824  38.827  38.828  38.829  41.107  46.215  46.216  46.217  46.218  55.290  55.291  55.292  55.293  55.294  57.900  57.901  57.902  57.903  57.905  57.906  57.907  57.908  57.909  57.910  57.911  57.912  57.913  57.916  62.972  571.308  651.084  651.085  651.086  681.865  681.866  681.867  681.868  46.88  46.89  H II 16  H II 16  Kleinschmidt 2467  Kleinschmidt 2468  Kleinschmidt 2476  Kleinschmidt 2477 | 1119  1131  70425 |
| Bremen |  | 000 | 152 | BR459 |
| Frankfurt | 6389  6390  6392  6393  6394 | 6343  6344  6345  6348  6349  6350  46105  46106  46107 | 6357  6359  6360  6361  6367  6392 | 6308  6395  6396  6399  6400  6401  6406  46086 |
| Hamburg | 1787  68229 | 1764  2961  52190  59780  68228  78222 |  |  |
| München | 18.90 | 14.882  17.801  18.307  18.566  19.227  20.543  20.544  27.239  27.240  172.227  172.231  191.358  191.361  191.363  18.91  1909/1312 | 18.858  18.859  172.242  172.243  172.244  172.245  172.246  172.254  182.109  182.490  182.491  182.493  182.494  182.545  182.546  182.548  57.52  60.21  71.2 | 122.114 |
| Paris | 485  588 | 282  2296  2304  1898-782 | 1265  2277  2278  1898-131 | 481  1232  2275  236065  236066 |
| Tring | 1937.10.14.333  1937.10.17.329  1937.10.17.330  1937.10.17.331  1937.10.17.332 | 1947.6.472  1965.m.11822  1965.m.11823  1965.m.11824  1965.m.11826  1965.m.11827  1965.m.11830  1965.m.11831  1965.m.11886  1965.m.11887  98.9.1.2448 |  |  |
| Wilhelmshaven |  |  | C5025 |  |
